# Educational Content and Acceptability of Training Using Mobile Instant Messaging in Large HIV Clinics in Malawi

**DOI:** 10.5334/aogh.3208

**Published:** 2021-04-16

**Authors:** Tom Heller, Sabine Bélard, Odala Sande, Tapiwa Kumwenda, Joe Gumulira, Prakash Ganesh, Salem Gugsa, Hannock Tweya, Sam Phiri

**Affiliations:** 1 Lighthouse Trust, Lilongwe, Malawi; 2 Department of Paediatrics, Division of Pneumonology and Immunology with intensive Medicine, Charité-Universitätsmedizin, Berlin, Germany; 3 Berlin Institute of Health, Berlin, Germany; 4 International Training and Education Center for Health, University of Washington, Seattle, WA, United States; 5 Department of Global Health, University of Washington, Seattle, United States; 6 The International Union Against Tuberculosis and Lung Disease, Paris, France; 7 Department of Medicine, University of North Carolina School of Medicine, Chapel Hill, NC, United States; 8 Department of Public Health, College of Medicine, School of Public Health and Family Medicine, University of Malawi, Malawi

## Abstract

**Background:**

In resource-limited settings, many HIV-infected patients with advanced HIV-related disease need specialized care not represented in guidelines. Training opportunities for healthcare providers on advanced HIV care are limited. The aim of this study was to evaluate the educational content and acceptability of mobile instant messaging (MIM) as a training and telemedicine tool for HIV care providers in Malawi.

**Methods:**

At the Lighthouse Clinic, Malawi, a MIM group using WhatsApp® was created for clinical officers and moderated by an infectious disease consultant. Questions encountered in the clinics as well as educational cases were posted; identifying data was not to be posted. MIM conversation was analyzed and in-depth interviews with users on its perceptions were performed.

**Results:**

MIM was utilized by 25 clinical officers and five physicians with an average of 2.3 threads/week over the observation period of 15 months. Discussed topics related to tuberculosis (25 threads), adverse drug reaction (22 threads), antiretroviral treatment (21 threads), cryptococcal meningitis (12 threads), and drug dosing/logistics. In 20% of the threads at least one image file was shared (mainly pictures of skin conditions and chest X-rays). In-depth interviews showed that clinical officers appreciated MIM group as a telemedicine consulting and training tool.

**Conclusion:**

MIM was a successful and well-accepted telemedicine tool for support and training of clinical officers providing HIV care in a resource-limited setting. MIM may be integrated in training strategies to expand the knowledge of HIV care providers.

## Introduction

In resource-limited settings HIV care follows a public health approach based on national and international guidelines and algorithms. Limited human resources and increasing patient numbers have required task-shifting with delegation of specific tasks from more skilled to less skilled health workers. In many public facilities in sub-Saharan Africa, nurses handle most patient visits and further task-shifting to non-clinical services is practiced [1].

An encouraging decline in the prevalence of advanced HIV disease at antiretroviral treatment (ART) initiation has been observed over recent years. However, a significant proportion of HIV patients still require complex management strategies due to severe immunosuppression, e.g., after late presentation or failing ART [2]. As a result, guidelines increasingly recommend differentiated care models for patients with advanced HIV infection [3]. Patients with complex conditions require more individualized management as they present with opportunistic infections, HIV-associated cancers, drug side effects, and high viral loads. Non-communicable diseases further complicate the long-term management of HIV patients [4].

In Malawi, clinical officers (COs) are the main healthcare workers providing services for patients with advanced HIV disease. COs are trained for three years at the College of Health Sciences and are responsible for the primary healthcare and selected higher care tasks of patients. Provision of HIV-specific training and support for these COs, who were not primarily educated in specific HIV-related medical expertise, is essential to ensure effective management of complex HIV patients. Training options on advanced HIV care are scarce in resource-limited settings, and the potential of remote telemedical support is increasingly recognized [5,6].

Mobile instant messaging (MIM) has seen tremendous growth during the past few years; the relatively simple communication technology allows real-time as well as asynchronous communication and sharing of images and video files. Furthermore, MIM supports group conversation, that is, communication between multiple participants in one digitally shared space. WhatsApp® is one of the popular platforms that offers these functions to more than 1.2 billion active users per month worldwide [7]. Free availability makes it particularly attractive in resource-limited settings. WhatsApp® is increasingly studied as an adjunctive tool of telemedicine and medical learning [8,9]. Reports describe its use by groups as disparate as English medical students [10], Indian orthopedic surgeons [11], and healthcare workers in rural Malawi exchanging medical information and knowledge [7].

This study describes the application and reception of a MIM group conversation for remote consultation and training on HIV-related patient conditions.

## Methods

### Setting

Lighthouse (LH) Trust is a Public Trust and WHO recognized Center of Excellence for integrated HIV prevention, treatment, care and support in Malawi. LH serves more than 45,000 ART patients at three urban sites in Lilongwe and Blantyre. Additionally, LH supports ART services in ten scattered urban and rural clinics in Lilongwe district, where over 50,000 further ART patients are treated. LH clinics provide patient-centered, integrated ART care including family planning, cervical cancer screening, hypertension screening and treatment, as well as treatment of sexually transmitted infections and Kaposi’s sarcoma. Patients are first attended by nurses then referred to one of the 21 COs providing further care if required by medical conditions.

### Consultation and training of COs by MIM

A MIM group messenger was created on WhatsApp® during an internal weekend workshop on clinical HIV case management. All COs working who had access to a smartphone were invited to the MIM group. The clinical advisor, an infectious disease and internal medicine physician, was part of the MIM group and served as a moderator.

All MIM group members were familiar with WhatsApp®, therefore no formal training was needed. In an initial 15-minute discussion, the following rules were agreed on: 1) all questions encountered in the clinics could be shared immediately to seek advice, 2) interesting or instructive cases should be shared for educational purposes, 3) to maintain patient confidentiality, patient names and images with patients faces were not to be posted (unless required and consented), and 4) posts should be restricted on medical topics and social communication should be limited as much as possible to limit unnecessary disturbance.

To stimulate discussions, the group moderator intermittently posted educational cases with questions that were discussed thereafter. The educational cases were chosen to cover rare, but important, clinical presentations of tuberculosis (TB) and HIV-associated conditions and were selected from the “Lighthouse training manual in HIV and TB medicine” [12].

To qualitatively assess the clinician’s perception and opinion of the MIM group, a semi-standardized in-depth interview was performed using a pre-defined questionnaire with 11 open-ended questions covering topics such as; importance and relevance of the MIM group for patient care, effectiveness of consultation and learning effects, reasons for participation or non-participation, and suggested improvements. Three COs from each of the three groups (“frequent participant”, “moderate participant” and “rare participant”) were randomly selected for interviews.

### Data Analysis

At the end of the project the complete communication was exported from WhatsApp® to a text file. The text was partitioned into individual threads. Each thread was coded with up to three summarizing key words. In addition, for each thread the member initiating the communication, number of communicating members, number of posts, and posted image or video files were assessed. For each CO, demographic and professional information was obtained and the number of threads she or he participated in was determined. To visualize the relative importance of the topics, a word cloud was generated from the assigned keywords using a freeware program named WordItOut [13].

### Ethics

The Malawi National Health Science Research Committee provides general oversight to Lighthouse clinics and granted approval (NHSRC Protocol # 829) for the routine collection and use of clinical and programmatic data for monitoring and evaluation, as was used in case of this study.

## Results

### Group Statistics

Data was analyzed from the MIM threads occurring between September 2016 to December 2017. At the end of the period the group had 30 members. Twenty-five members were COs (four joined the MIM group in the month before the analysis) and five were physicians (the moderator, an additional clinical advisor, the medical manager of LH, and two external physicians based in Holland and Germany, both of whom had been working at LH before).

**Figure 1:**
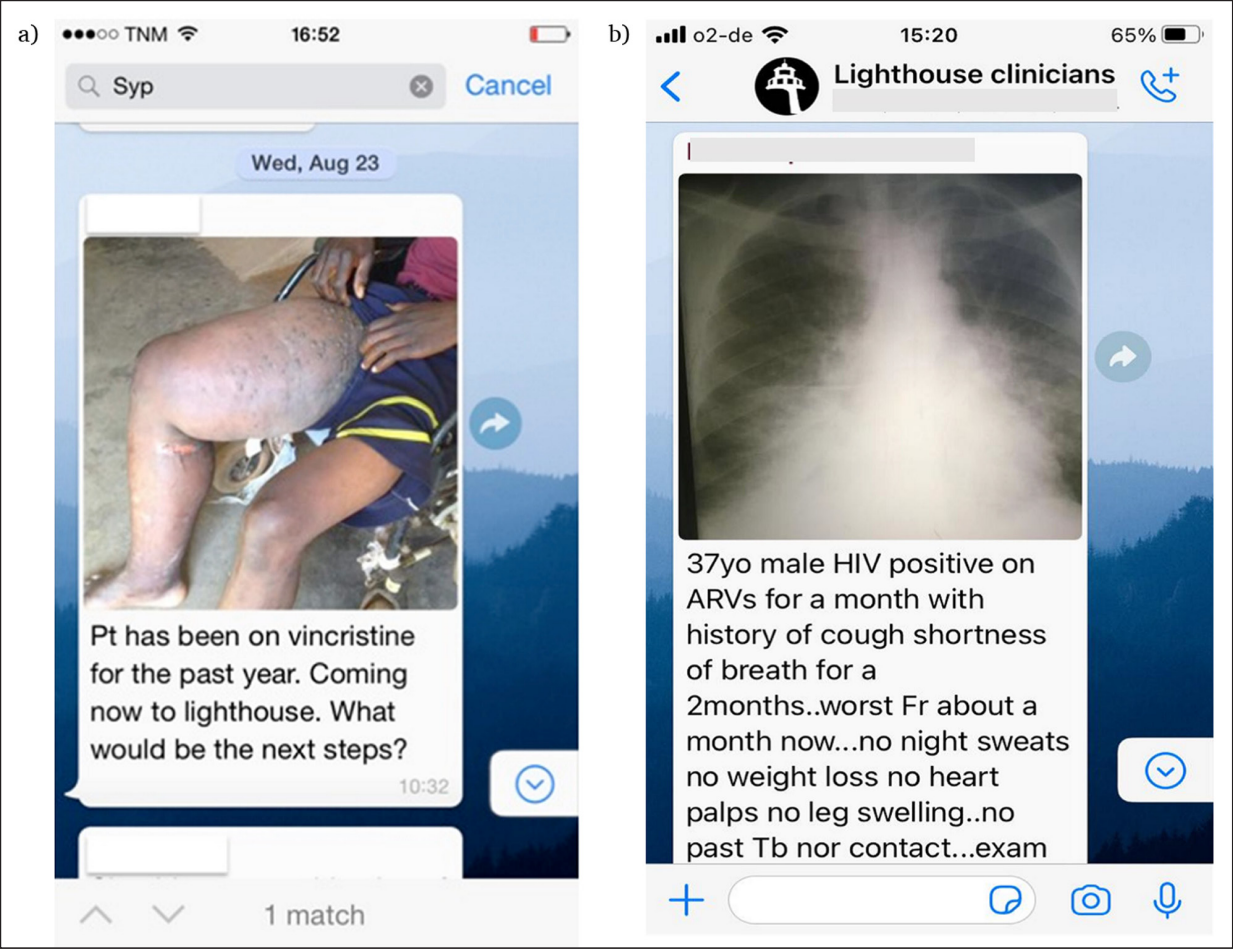
Examples of posts that include image files.

The communication contained 143 threads (average 2.3/week) and 1733 individual posts. The median number of posts per thread was 10 (min-max 1–52), the median number of participants was 4 (min-max 1–16). In 29 of the threads (20.3%), at least one image file was shared. Clinical pictures were primarily of skin conditions and were included in 15 (10.5%) of the threads (***Figure 1a***); chest x-ray images were shared in 12 (8.3%) and ultrasound files in 4 (2.8%) (***Figure 1b***) threads. The ultrasound files were created by filming the screen and transmitting the video, the image quality was adequate to recognize the pathology.

The participating COs were grouped into three groups: *frequent* participants posted in more than 30 threads, *moderate* participants posted in 10–29 threads and *rare* participants posted in less than 10 threads. Only one participant did not post at all. The groups are summarized in ***Table 1***. The moderator initiated 39 (27%) and participated in 93 (65%) threads. In 24 (17%) threads training material was shared, which included presentations, journal articles, material from weekend trainings, and educational cases.

**Table 1:** Characteristics of clinical officers participating in the Lighthouse MIM group.


PARTICIPATION IN THREADS	FREQUENT >30	MEDIUM 29–10	RARE <10

Total participants, n*	9	6	6

Gender (M/F), n	8/1	5/1	5/1

Age, years (mean, SD)	37.4 ± 5.8	38 ± 3.9	42 ± 8.0

Years of experience in HIV (mean, SD)	9.6 ± 2.8	6.6 ± 3.8	11.8 ± 2.8

Participation in threads, n (median, min-max)	40 (31–51)	14.5 (14–29)	6.5 (0–9)

Initiation of threads, n (median, min-max)	6 (1–18)	2 (0–4)	1 (0–4)


* The medical doctors and the recently joined clinical officers were excluded from analysis.

### Thematic Analysis

**Figure 2:**
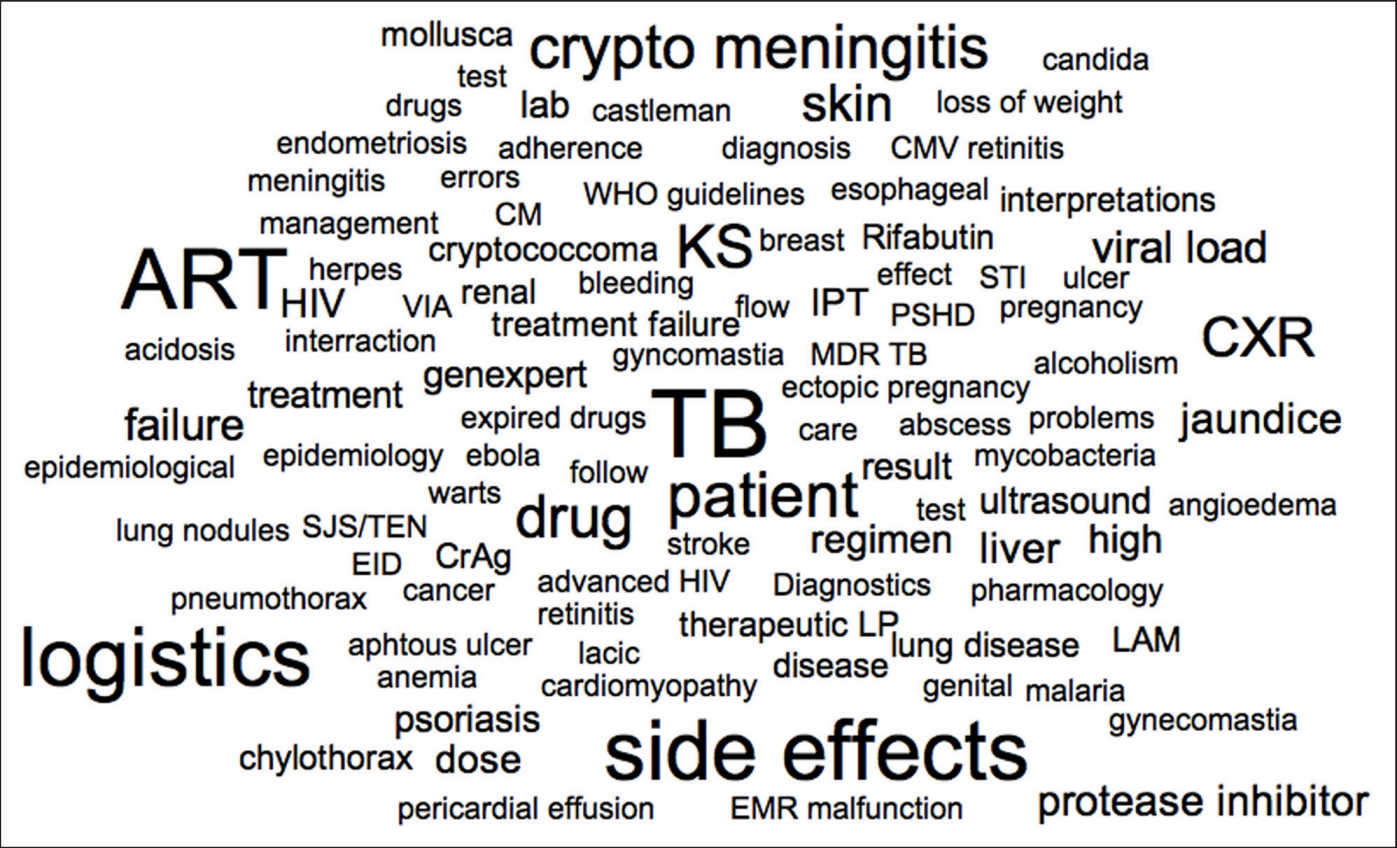
Word cloud visualizing the thematic key words of the WhatsApp® group discussions.

Analysis of thread-linked key words showed that 22 (15.3%) of threads mainly contained administrative information (e.g. group administration, rotation, and training schedules). After excluding these, the remaining 253 key words were analyzed. Topics discussed most frequently were related to TB (25 threads), adverse drug reactions (22), and ART (21). All topics featured in 10 or more threads are shown in ***Table 2***; all key words are visualized in the thematic word cloud presented in ***Figure 2***.

**Table 2:** Thematic key words discussed in more than 10 threads in the LH CO MIM group.


TOPIC	FREQUENCY (n)

Tuberculosis	25

Side effects	22

ART	21

Cryptococcal meningitis	12

Drug dosing or drug logistics	12

Kaposi’s sarcoma	11

ART failure or high viral load	10

Radiograph analysis	10

Lab results or lab logistics	10


### Qualitative assessment

Nine COs (30% of all group members) were interviewed about their opinion and experiences with the MIM group; their answers are summarized thematically below.

#### 1) Perceived importance and relevance of the MIM group for clinical work

All interviewed COs affirmed that topics and cases discussed were important and represented the issues they encounter in their daily practice.

… because most of times, I think 99% of the times, the kind of cases that are discussed are cases which we see in the clinic…. So, it is really relevant to us. (Frequent participant, 3)

The participants used the MIM group for different purposes including administrative communication (e.g., meeting times, clinic rotation). However, the main use described by participants was for learning purposes, specifically to get feedback from fellow COs on how to manage patients.

Like if I have a case that I am stuck with, I post on the group, I get suggestions and I can easily do several things pertaining to the suggestions given and again if it requires referral I could be advised to refer the patient to such-and-such center for such-and-such intervention, that’s how it makes life easier. (Frequent participant, 3)

#### 2) Perceived training effect of MIM group case discussions and shared material

The COs reported that the MIM group helped them to learn and improve their clinical HIV skills. Most COs recalled more than one of the cases that were presented in the MIM group. Topics easily recalled included TB focused point-of-care ultrasound, isoniazid side effects, jaundice, liver cases, TB, and HIV cases.

The cases which COs recalled easily were those that helped them directly manage patients.

Because they helped clinical management in patients, because sometimes you learn from those ideas people are putting forward, so you learn a lot and if you have a patient in front of you then you manage that patient well. (Moderate participant, 3)

COs also mentioned topics that they would want to be posted and discussed more often. The most frequently mentioned topic was “HIV/TB”. More specific topics COs would like to see being discussed were “3^rd^ line ART”, “ART side effects”, “pediatric ART”, “Kaposi’s sarcoma” and “hepatic side effects”.

Educational learning cases were generally appreciated as long as they “were close to reality”. They were felt to help stimulate learning.

They were good cases… in the sense that [they] were not straight forward cases, they needed one to think or to do some research on what’s going on, so… it was good. (Frequent participant, 3)

Additionally, there was understanding that not every topic could be presented by a real case, so the educational cases were good to fill in that gap.

… the educational cases actually they are good because you will not a have real case for every condition, so the educational cases will actually give room to fill in for that deficiency… it appears to give room for mental activation, discussion around the topic, it might sound abstract but it… gives an opportunity to be prepared for a case that probably we have never seen when it comes around. (Frequent participant, 1)

#### 3) Reasons for “non-active” participation

Moderate and rare participants cited discomfort in group discussions and lack of familiarity in the topics discussed as the potential reasons for not participating in the group discussion.

Maybe they are not comfortable as they feel like they are not experienced enough to comment on the issues … (Moderate participant, 3)

… maybe some of us do not read much and are shy. (Rare participant, 2)

One logistical reason cited was limited access to a smart phone.

It is challenging for others to comment, because they use other people’s phones. (Moderate participant, 3)

COs, even those with few postings, explained to have been “active followers” as they were checking messages in the MIM group on a daily basis.

… I check every time and I follow what people are discussing. (Moderate participant, 3)

Those who did not participate frequently reported to have posted cases and even received feedback from colleagues, which helped motivate and make them feel part of the group.

#### 4) Perceived intrusion into private life and comparison of the project’s MIM group to other work-related MIM groups

Generally, COs shared the common view that the messages posted in the group after working hours were not perceived as problematic. Very few said that messages posted after working hours intruded on their personal time but emphasized that it did not happen frequently.

Yes, I find the messages intruding after working hours but since they are not much, therefore to me it is not a problem. (Moderate participant, 2)

Many COs are members of other work-related MIM groups apart from the MIM group of this project; other groups were named the “adolescent management group”, “infection prevention group” and the “quality improvement group”. When given a chance to compare, the above point was further reinforced.

Yes, the others are social and noisy while this is educative and straight to important things. (Frequent participant, 1)

The MIM group of this project was perceived to be for professional use only and for discussing complex clinical issues. The input from different local and international advisors was acknowledged as positive.

## Discussion

A clinical consultation and training group using MIM was successfully established at LH and was appreciated by the participants. The thematic analysis reflects very well the diseases and related care problems common in the project’s setting. The majority of COs participated actively in the group and even participants not posting cases or comments benefited by passively following the discussion of others.

The MIM group was perceived as an important tool to obtain timely advice from colleagues on physically present patients. As such, it constitutes a “telemedicine system” and it is prudent to consider some of the issues on telemedicine suggested by the World Health Organization [14].

### Costs for software and hardware

Costs for the software are negligible as WhatsApp® (and other MIM messengers) is widely available and used for social purposes. Comparatively cheap data bundles are available from local mobile network providers. Nevertheless, as seen in our study, COs without smartphones are excluded or at least have reduced access to the MIM group, pointing towards access disadvantage for the poorest.

### Local bandwidth and image quality

MIM requires relatively small bandwidth and the local 3G system was sufficient for successful use. The transfer of images and video clips was feasible, quick, and aided in clinical diagnosis. The image quality of chest radiographs and video clips of ultrasound were sufficient to allow diagnostic conclusions. This is in accordance with previous studies, finding strong agreement in the assessment of radiographs and CT scans sent via WhatsApp® e.g. for assessment of tibial fractures [15].

### Technical expertise and legalities

Remote consultation, even more so when cross-border cooperation is involved, raises a question of legal responsibilities; this issue is currently not sufficiently addressed in health laws of many countries, [14] nor is it in Malawi. Currently the responsibility lies mostly with the directly treating CO. In international collaborations it is important that the advising consultant is familiar with local protocols to be able to suggest feasible and appropriate diagnostic and therapeutic advice. In our group all external advising members were aware of the locally available resources as they worked with LH previously.

### Patient confidentiality/privacy

WhatsApp® uses end-to-end encryption of all message formats across all operating systems, [16] therefore data security seems maintained. Nevertheless, it is a relevant concern that confidentiality breaches could occur, and patient data could be leaked. Strict implementation of rules like the use of initials only and avoiding posting pictures of the face helped ensuring confidentiality in our project. De-identifying patient information bears potentially dangerous consequences as patients are treated between multiple clinicians and patients may be confounded. [16] In instances where names needed to be exchanged to identify patients (e.g. those with cryptococcal meningitis), offline communication of patient identifiers was encouraged.

Besides its usefulness as a telemedicine consulting tool, the MIM group was appreciated as a learning tool. Participant interviews also suggested that such groups can be used to identify areas that require discussion and inform training planning for weekend face-to-face trainings. Clinicians remembered specific cases that were discussed in the MIM group; therefore, a learning effect can be postulated. Our findings are in line with previous reports suggesting MIM as a valuable tool to facilitate training and learning in various topics. It was found to enhance critical thinking in students of physics, [17] but also stimulated learning in medical students in topics such as pathology [9] and community medicine [18]. One commonly mentioned concern is that MIM, with constant availability, interferes with clinicians resting times. This was not felt as a problem by the participants in the LH group; rules that minimize “social noise” allowed us to limit intrusiveness into the private time of the clinical staff which was perceived as “professional”. Limitations of this report include the limited sample size of participants and possible selection bias.

Training HIV care providers in resource-limited settings without depleting the workforce by long absenteeism due to attending trainings is a challenge. Blended learning methods including distance learning, remote consultations, and case conferences are recognized as training options and can minimize the impact of in-service training on service delivery [19,20]. MIM can be an essential component of blended method learning courses for advanced HIV care; if it is used to supplement face-to-face teachings and guided remote learning.

## Conclusion

MIM for consultation of COs in HIV care was considered successful as a telemedicine as well as a learning tool and was appreciated by the participants. MIM can be recommended as a component of blended-method learning for HIV care to expand ART knowledge through remote advice in resource-limited settings.

## References

[1] Tweya H, Ben-Smith A, Weigel R, et al. ‘Task shifting’ in an antiretroviral clinic in Malawi: can health surveillance assistants manage patients safely? Public Health Action. 2012; 2: 178–180. DOI: 10.5588/pha.12.0018 26392980 PMC4463055

[2] Auld AF, Shiraishi RW, Oboho I, et al. Trends in prevalence of advanced HIV disease at antiretroviral therapy enrollment—10 countries, 2004–2015. MMWR Morbidity and Mortality Weekly Report. 2017; 66: 558. DOI: 10.15585/mmwr.mm6621a3 28570507 PMC5657820

[3] World Health Organization. Guidelines for managing advanced HIV disease and rapid initiation of antiretroviral therapy. Geneva; 2017. 29341560

[4] Guaraldi G, Orlando G, Zona S, et al. Premature age-related comorbidities among HIV-infected persons compared with the general population. Clinical Infectious Diseases. 2011; 53: 1120–6. DOI: 10.1093/cid/cir627 21998278

[5] Chung MH, Severynen AO, Hals MP, Harrington RD, Spach DH, Kim HN. Offering an American graduate medical HIV course to health care workers in resource-limited settings via the Internet. PloS one. 2012; 7: e52663. DOI: 10.1371/journal.pone.0052663 23285139 PMC3527561

[6] Zolfo M, Lynen L, Dierckx J, Colebunders R. Remote consultations and HIV/AIDS continuing education in low-resource settings. International Journal of Medical Informatics. 2006; 75: 633–637. DOI: 10.1016/j.ijmedinf.2006.03.002 16647877

[7] Pimmer C, Mhango S, Mzumara A, Mbvundula F. Mobile instant messaging for rural community health workers: a case from Malawi. Global Health Action. 2017; 10: 1368236. DOI: 10.1080/16549716.2017.1368236 28914165 PMC5645652

[8] Giordano V, Koch H, Godoy-Santos A, Belangero WD, Pires RES, Labronici P. WhatsApp messenger as an adjunctive tool for telemedicine: an overview. Interactive Journal of Medical Research. 2017; 6: 2. DOI: 10.2196/ijmr.6214 PMC5544893 28733273

[9] Gon S, Rawekar A. Effectivity of e-learning through WhatsApp as a Teaching Learning Tool. MVP Journal of Medical Sciences. 2017; 4: 19. DOI: 10.18311/mvpjms/0/v0/i0/8454

[10] Raiman L, Antbring R, Mahmood A. WhatsApp messenger as a tool to supplement medical education for medical students on clinical attachment. BMC Medical Education. 2017; 17: 7. DOI: 10.1186/s12909-017-0855-x 28061777 PMC5219809

[11] Choudhari P. Study on effectiveness of communication amongst members at department of orthopedics surgery unit 3 using smartphone and mobile WhatsApp. International Surgery Journal. 2016; 1: 9–12. DOI: 10.5455/2349-2902.isj20140504

[12] Heller T. Lighthouse training manual in HIV and TB medicine, Lighthouse, Lilongwe, Malawi, mwlighthouse.org/files/download/02a7f94bdc99d37. Accessed on 11.5.2018.

[13] WordItOut. https://worditout.com. Accessed on 01.05.2018.

[14] World Health Organization. Global Observatory for eHealth Series-volume 2: Telemedicine. Opportunities and Developments in Member States. Geneva, Switzerland. 2011; 8–23.

[15] Giordano V, Koch HA, Mendes CH, Bergamin A, de Souza FS, do Amaral NP. WhatsApp Messenger is useful and reproducible in the assessment of tibial plateau fractures: inter-and intra-observer agreement study. International Journal of Medical Informatics. 2015; 84: 141–148. DOI: 10.1016/j.ijmedinf.2014.11.002 25468642

[16] Mars M, Scott RE. WhatsApp in Clinical Practice: A Literature review. Studies in Health Technology and Informatics. 2016; 231: 82–90. 27782019

[17] Kustijono R, Zuhri F. The use of Facebook and WhatsApp application in learning process of physics to train students’ critical thinking skills. IOP Conference Series: Materials Science and Engineering. 2018; 296: 1. IOP Publishing, 2018. DOI: 10.1088/1757-899X/296/1/012025

[18] Dyavarishetty PV, Dipak CP. An interventional study to assess the effectiveness of ‘WhatsApp’ as a teaching learning tool in community medicine. International Journal of Community Medicine and Public Health. 2017; 4: 2564–2569. DOI: 10.18203/2394-6040.ijcmph20172860

[19] Clevenbergh P, Van der Borght SF, Van Cranenburgh K, et al. Database-supported teleconferencing: an additional clinical mentoring tool to assist a multinational company HIV/AIDS treatment program in Africa. HIV Clinical Trials. 2006; 7: 255–262. DOI: 10.1310/hct0705-255 17162320

[20] McCarthy EA, O’Brien ME, Rodriguez WR. Training and HIV-treatment scale-up: establishing an implementation research agenda. PLoS Medicine. 2006; 3: e304. DOI: 10.1371/journal.pmed.0030304 16792434 PMC1483909

